# TRIM29 inhibits miR-873-5P biogenesis via CYTOR to upregulate fibronectin 1 and promotes invasion of papillary thyroid cancer cells

**DOI:** 10.1038/s41419-020-03018-3

**Published:** 2020-09-29

**Authors:** Tong Wu, Da-Lin Zhang, Jia-Mei Wang, Jing-Yi Jiang, Xin Du, Xiao-Yan Zeng, Zhen-Xian Du

**Affiliations:** 1 Department of Endocrinology & Metabolism, the 1st affiliated Hospital, China Medical University, 110001 Shenyang, China; 2 Department of Thyroid Surgery, the 1st affiliated Hospital, China Medical University, 110001 Shenyang, China; 3 Department of Laboratory Medicine, the 1st affiliated hospital, China Medical University, 110001 Shenyang, China; 4 Department of Biochemistry & Molecular Biology, China Medical University, 110122 Shenyang, China

**Keywords:** Cancer microenvironment, Endocrine cancer

## Abstract

Papillary thyroid cancer (PTC) is the most common endocrine tumor with an increasing incidence, has a strong propensity for neck lymph node metastasis. Limited treatment options are available for patients with advanced or recurrent metastatic disease, resulting in a poor prognosis. Tripartite motif protein 29 (TRIM29) is dysregulated in various cancer and functions as oncogene or tumor suppressor in discrete cancers. In this study, we found that both TRIM29 and fibronectin 1 (FN1) were upregulated with positive correlation in PTC tissues. Neither overexpression nor downregulation of TRIM29 altered the proliferation of PTC cells significantly. Overexpression of TRIM29 significantly promotes, while knockdown of TRIM29 significantly decreases migration and invasion by regulating FN1 expression in PTC cells. In terms of mechanism, we found that TRIM29 altered the stability of FN1 mRNA via regulation of miR-873-5p expression. The current study also demonstrated that long non-coding RNA (LncRNA) CYTOR suppressed maturation of miR-873-5p via interaction with premiR-873, and TRIM29 decreased miR-873-5p via upregulation of CYTOR. This study suggests that involvement of TRIM29 in migration and invasion in PTC cells may reveal potential metastatic mechanism of PTC and represent a novel therapeutic target and strategy.

## Introduction

Thyroid cancer (TC) is the most frequent endocrine cancer, the appreciable rising incidence rates have been reported globally across multiple epidemiologic studies over the last few decades^1–3^. It is already the sixth most common cancer among women in the United States^4^. Thyroid cancers can be classified into four types: papillary carcinoma, follicular carcinoma, medullary carcinoma, and anaplastic carcinoma, while papillary thyroid cancer (PTC) is the most common histotype, accounting for ~85–90% of all^5,6^. Most patients have a favorable prognosis through surgical removal, thyroid hormone, and adjuvant radioactive iodine therapy, and the survival is in fact extremely good over 98% 5-year survival rate in the United States^4^. Nevertheless, PTC has a strong propensity for neck lymph node metastasis, it involves cervical lymph node metastases in at least 20–50% of patients^7,8^. There is also substantial impact on tumor recurrence and scope of surgical resection, although local invasion occurs in a minority of patients^9,10^. An evidence from a large-scale nested case–control study indicated that lymph node metastasis and incomplete surgical excision are the two primary factors responsible for higher mortality^11^. Furthermore, patients with distant metastatic disease are less common with rates between 3 and 15%^12,13^, but merely have a 5-year survival rate as low as ~50%^14^. Thus, it is urgent to identify the potential molecular mechanisms underlying PTC metastatic behavior.

Tripartite motif-containing (TRIM) family is composed of several evolutionary conserved domains, including a RING finger, one or two B-box zinc-finger motifs (B1 and B2) and a coiled-coil region (RBCC), and involved in broad range of physiological and biological processes intracellular signaling, such as cell development, apoptosis, protein quality control, and carcinogenesis^15–17^. Among the TRIM protein members, TRIM29 is dissimilar to others because it has the B1-B2-CC domains but lacks the RING domain which is common for TRIM family proteins^15^. Accumulating studies have indicated that TRIM29 could regulate tumorigenesis positively and negatively possibly dependent on tissue/cell contexts. TRIM29 has been found to be upregulated and associated with tumor progression and poor prognosis in cervical cancer^18^, pancreatic cancer^19^, gastric cancer^20^, and colorectal cancer^21^. On the contrary, TRIM29 functions as a tumor suppressor in hepatocellular carcinoma^22^ and breast cancer^23^. Despite TRIM29 plays both promotive and suppressive roles in different cancers, a previous study reported that TRIM29 was upregulated and correlated with poor prognosis in TC patients, and functions as a oncogene via P13K/AKT signaling pathway^24^, another study observed that TRIM29 regulated cell growth via LncRNA HOXA11-AS/miR-761/TRIM29 axis^25^. Considering that TRIM29 is a unique multifunctional TRIM protein involved in many other signaling pathways such as Wnt/beta-catenin and TWIST, it is necessary to further verify other biological function and molecular mechanism especially in PTC.

Long non-coding RNAs (LncRNAs) represent a huge class of RNAs with limited protein-coding potential that are more than 200 nucleotides in length and lack of detectable open reading frame (ORF), typical initiation codon, 3-untranslated regions (UTRs), and termination codon^26,27^. The function of LincRNAs is closely related to sub-cellular distribution. LncRNAs are well known to act as the endogenous microRNA sponge to regulate the microRNA targets in the cytoplasm^28,29^. Cytoskeleton regulator (CYTOR, or long intergenic non-coding RNA 00152) has been demonstrated to regulate gene expression by various mechanisms as a crucial oncogene in many kinds of cancers excluding PTC^30–32^. Studies showed that LncRNA CYTOR could act as ceRNA in cytoplasm or bind to polycomb repressive complex 2 (PRC2) to epigenetically regulate genes’ expression in nucleus^30,32^.

In this study, we identified that TRIM29 upregulated LncRNA CYTOR, which interact with premiR-873 to inhibit miR-873-5p biogenesis. Subsequently, inhibition of miR-873-5p promoted invasion of PTC cells via upregulating FN1, which has been confirmed that was highly expressed in tumor tissues and promotes proliferation, adhesion, and migration of PTC cells in vitro^33^. These findings revealed an unidentified role of TRIM29 in suppression of miR-873-5p maturation via upregulation of CYTOR. In summary, our study displayed a novel mechanism about PTC progression mediated by TRIM29 and provided a promising treatment strategy.

## Materials and methods

### Cell culture

Human papillary thyroid cancer cell lines, K1, TPC1, and IHH4 were cultured. The K1 cell lines was purchased from the European Collection of Authenticated Cell Culture (ECACC, UK). IHH4 was obtained from the Health Science Research Resources Bank (Osaka, Japan). The TPC1 cell line was a gift from Professor Meiping Shen (Department of General Surgery, The First Affiliated Hospital of Nanjing Medical University, Nanjing, Jiangsu). K1 cells were maintained in Dulbecco’s modified eagle’s medium (DMEM): Ham’s F12: MCDB 105 (2:1:1) and 2 mM glutamine supplemented with 10% FBS. IHH4 cells were maintained in a 1:1mixture of RPMI-1640 and DMEM supplemented with 10% FBS. TPC1 cells were maintained in DMEM with 10%FBS. The cells were maintained in a humidified atmosphere at 37 °C with 5% CO2.

### Tissue specimens and ethics statement

A total of 84 PTC tissues and corresponding noncancerous thyroid tissues were collected from patients who underwent surgical resection at the first affiliated hospital of China Medical University. The diagnosis of PTC was pathologically confirmed either intra- or postoperatively. Written informed consent was obtained from all patients and this study was approved by the Ethics Committee of China Medical University and complied with the Declaration of Helsinki. All specimens were frozen in liquid nitrogen immediately and stored at −80 °C until use.

### Western blotting

Tissues and Cells were washed with ice-cold PBS and were extracted using a lysis buffer (20 mM Tris-HCl, 150 mM NaCl, 2 mM EDTA, 1%NP-40, and protease inhibitor cocktail) on ice. Extracted proteins were quantified using the BCA protein assay kit (Thermo Scientific, 23225). Equivalent amounts of protein (20 μg) were separated using 7% SDS-PAGE and transferred to PVDF membranes. After blocking in 5% non-fat milk for 2 h, the membranes were incubated overnight at 4 °C with primary antibodies recognizing TRIM29 (1:1000 dilution; Sigma-Aldrich, USA), FN1 (1:500 dilution; Abcam, UK), and GAPDH (1:1000 dilution; Proteintech, USA). After incubation with secondary antibodies (1:5000 dilution; Jackson ImmunoResearch, PA, USA), the protein bands were visualized by chemiluminescence using a GE Amersham Imager 600 (GE, USA).

### RNA extraction and quantitative real-time RT-PCR

Total RNA from tissues were extracted using Trizol and total RNA from cells were extracted using RNeasy^®^ Mini Kit (250, QIAGEN, GER) according to the manufacturer’s instructions. RNA was reversely transcribed to cDNA using a PrimeScript RT reagent kit (Takara, Dalian, China). Real-time RT-PCR was performed using SYBR Premix Ex Taq^TM^ II (Takara, Dalian, China) on an ABI 7500 system (Applied Biosystems, Foster City, CA, USA).

### Determination of mRNA half-life

To measure the half-life of endogenous FN1 mRNA, actinomycin D was added into the cell culture medium and total RNA was prepared at the times indicated and subjected to qRT-PCR analysis using specific primers. mRNA levels were normalized to 18S rRNA and plotted as a percentage of the value at time zero (set at 100%).

### Label and capture nascent RNA

Newly synthesized RNA was labeled and isolated using Click-iT Nascent RNA Capture kit (Invitrogen) according to the manufacturer’s instruction. In brief, cells were incubated with 0.2 mM of 5-ethymyl uridine for 4 h to label nascent RNA. Total RNA was isolated using Trizol reagent, and the 5-ethymyl uridine–labeled nascent RNA was biotinylated in Click-iT reaction buffer with 0.5 mM of biotin azide. The biotinylated nascent RNA was subsequently captured on streptavidin magnetic beads.

### RNA immunoprecipitation

Magna RIP^TM^ RNA-binding protein immunoprecipitation kit (Millipore) was used for RNA immunoprecipitation (RIP) procedures according to the manufacturer’s protocol. Ago2, TRBP, Dicer, and Flag antibody was used to pull down FN1 mRNA and premiR-873. After the antibody was recovered by protein A/G beads, standard qRT-PCR was performed to detect FN1 mRNA and premiR-873 in the precipitates.

### Electrophoretic mobility shift assay for RNA–RNA interaction

Interaction between CYTOR and premiR-873 was investigated using LightShift® Chemiluminescent RNA EMSA Kit according to the manufacturer’s instruction. Briefly, 10 ng/μl biotin-labeled premiR-873 and 100 ng/μl CYTOR fragment with potential to interact with premiR-873 were incubated in binding mixture at room temperature for 30 min and followed by electrophoresis and detection biotin-labeled RNA by chemiluminescence as the kit’s protocol.

### Fluorescence in situ hybridization staining

Fluorescence in situ hybridization (FISH) was performed to investigate distribution of lncRNA CYTOR according to the manufacture’s instructions (Histology FISH Accessory Kit, Agilent). Briefly, the cells were fixed with 4% paraformaldehyde at 10 min at room temperature and permeabilized in PBS containing 0.2% Triton-100 for 5 min at 4 °C. Hybridization was carried out with a FISH probe specific to CYTOR in a moist chamber at 37 °C in the dark overnight. Fluorescent signals were visualized using an Olympus fluorescence microscope.

### Immunofluorescence

IHH4 cells were fixed with 4% paraformaldehyde for 20 min at room temperature and permeabilized with 10% FBS in PBS containing 0.2% Triton X-100 for 30 min after sufficient washing. The cells were incubated with the primary antibody against TRIM29 for 1 h, subsequently subjected to staining with Alexa Fluor Plus second antibody. Finally, the images were acquired using an Olympus fluorescence microscope.

### Transwell invasion and migration assays

The transwell system (BD Biosciences, San Jose, CA, USA) were employed for cell migration and invasion assays. Transwell inserts coated with Matrigel on the upper layers were used for invasion assay. Uncoated inserts were used for migration assay. Briefly, cells were seeded into the upper chamber with FBS-free medium, and lower chamber was filled with full medium. The cells were incubated in a humidified 5% CO2 incubator at 37 °C for 24 h. The invaded or migrated cells were fixed in 4% paraformaldehyde for 5 min and then stained with 0.3% crystal violet. Invading cells or migrating cells were counted under a light microscope.

### Luciferase reporter assay

The DNA fragment of 3′ untranslational region (UTR) of human FN1 was PCR amplified and cloned downstream of a firefly luciferase gene in pGL4 vector. Cells were co-transfected with 4 μg of control or reporter DNA, and 0.2 μg of Renilla luciferase (pRL-TK) as a normalizing control. Luciferase activity was determined using a Dual-Luciferase Reporter Assay (Promega) according to the manufacturer’s instructions 48 h after transfections. Firefly luciferase was normalized against Renilla luciferase. Transfections were performed in triplicates and repeated three times.

### Statistics

The statistical significance of the difference was analyzed by ANOVA and post hoc Dunnett’s test. Statistical significance was defined as *P* < 0.05. All experiments were repeated three times, and data were expressed as the mean ± SD (standard deviation) from a representative experiment.

## Results

### TRIM29 affects migratory and invasive capacities, but not the proliferation of PTC cells

**Fig. 1:**
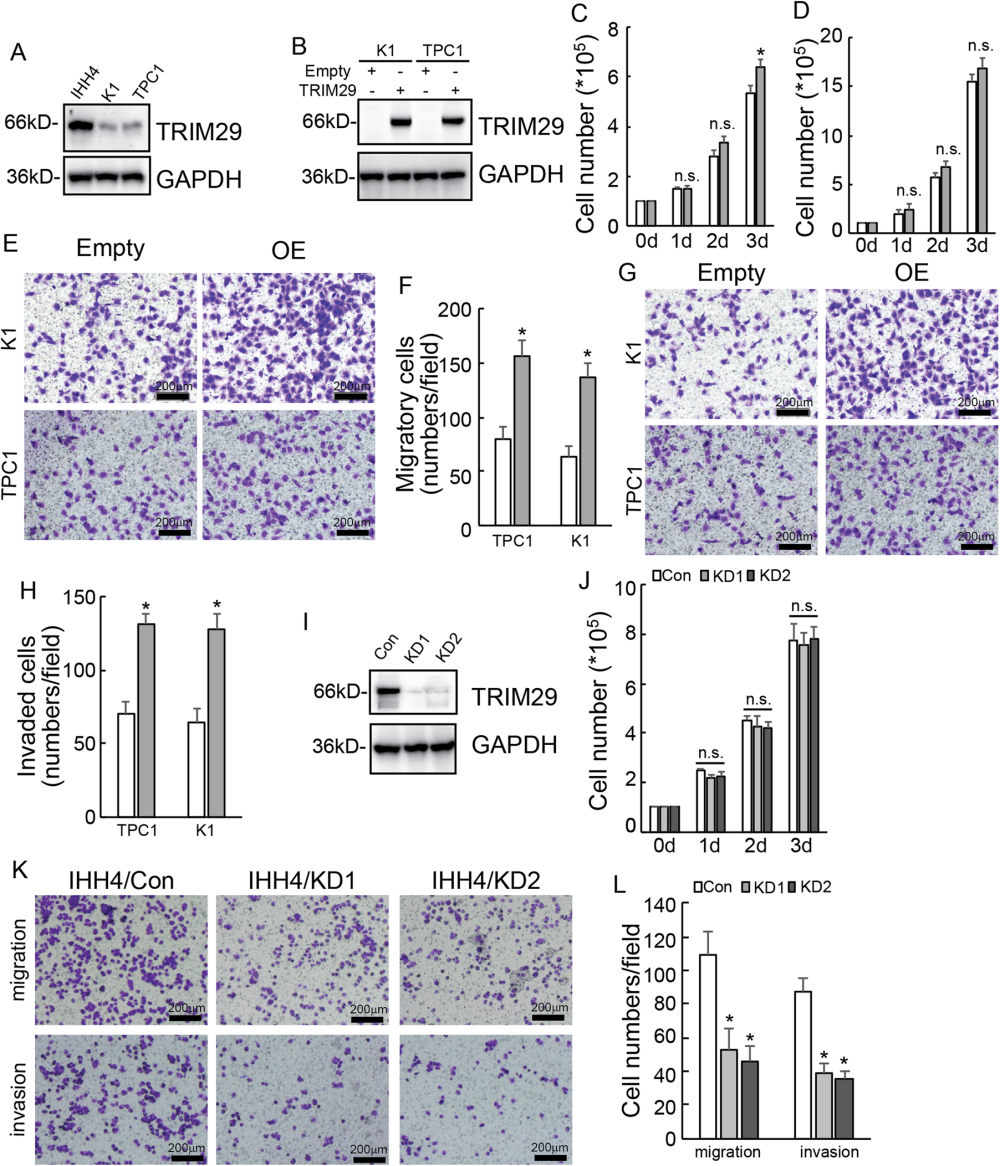
Promotive or suppressive function on migration and invasion by TRIM29 overexpression or knockdown in PTC cells, respectively. **a** TRIM29 endogenous expression was investigated using western blot analysis in a panel of PTC cells. **b** K1 and TPC1 were infected with lentivirus containing empty or TRIM29 construct, TRIM29 expression was confirmed by western blot analysis. **c**, **d** Control and TRIM29 overexpression K1 (**c**) and TPC1 (**d**) cells were plated on 6-well, and viable cell numbers were counted daily for 3 days. **e**, **f** The migration of control or TRIM29 overexpression K1 and TPC1 cells were evaluated by Matrigel-uncoated Transwell. Representative photographs were presented (**e**) and their numbers were counted (**f**). **g**, **h** The invasion of control or TRIM29 overexpression K1 and TPC1 cells were evaluated by Matrigel-coated. Transwell Representative photographs were presented (**g**) and their numbers were counted (**h**). **i** IHH4 cells were infected with gRNA guided TRIM29 using CRISPR/Cas9 system, western blot was performed using the indicated antibodies. **j** Control and TRIM29 knockdown IHH4 cells were plated on six-well, and viable cell numbers were counted daily for 3 days. **k**, **l** The migration and invasion of control or TRIM29 knockdown IHH4 cells were evaluated by Matrigel-uncoated or Matrigel-coated Transwell, Representative photographs were presented (**k**) and their numbers were counted (**l**).The data are presented as the mean (SD) of three repeated experiments. *P < 0.05, n.s. not significant.

We assessed TRIM29 endogenous expression in three PTC cell lines (IHH4, K1, and TPC1) and found that a relatively lower expression level of TRIM29 in K1 and TPC1 cells, while a relatively higher expression level in IHH4 cells (Fig. 1a). To explore the potential functions of TRIM29 in PTC cells, TRIM29 was ectopically overexpressed in K1 and TPC1 cells (Fig. 1b), which had relatively low endogenous TRIM29 expression. In general, viable cell count assessment showed that TRIM29 overexpression had no effects on proliferation of K1 (Fig. 1c) and TPC1 (Fig. 1d) cells, despite some differences in K1 cell counts on the third day. Transwell assays demonstrated that TRIM29 overexpression significantly promoted migration (Fig. 1e, f) and invasion (Fig. 1g, h) of both K1 and TPC1 cells. TRIM29 was also knocked down in IHH4 cells using CRISPR-Cas9 system, two different gRNAs specific against TRIM29 significantly decreased TRIM29 expression (Fig. 1i). Knockdown of TRIM29 also demonstrated no obvious effects on proliferation (Fig. 1j), while significantly suppressed migration and invasion (Fig. 1k, l) of IHH4 cells.

### TRIM29 affects invasive capacities of PTC cells by regulating FN1

**Fig. 2:**
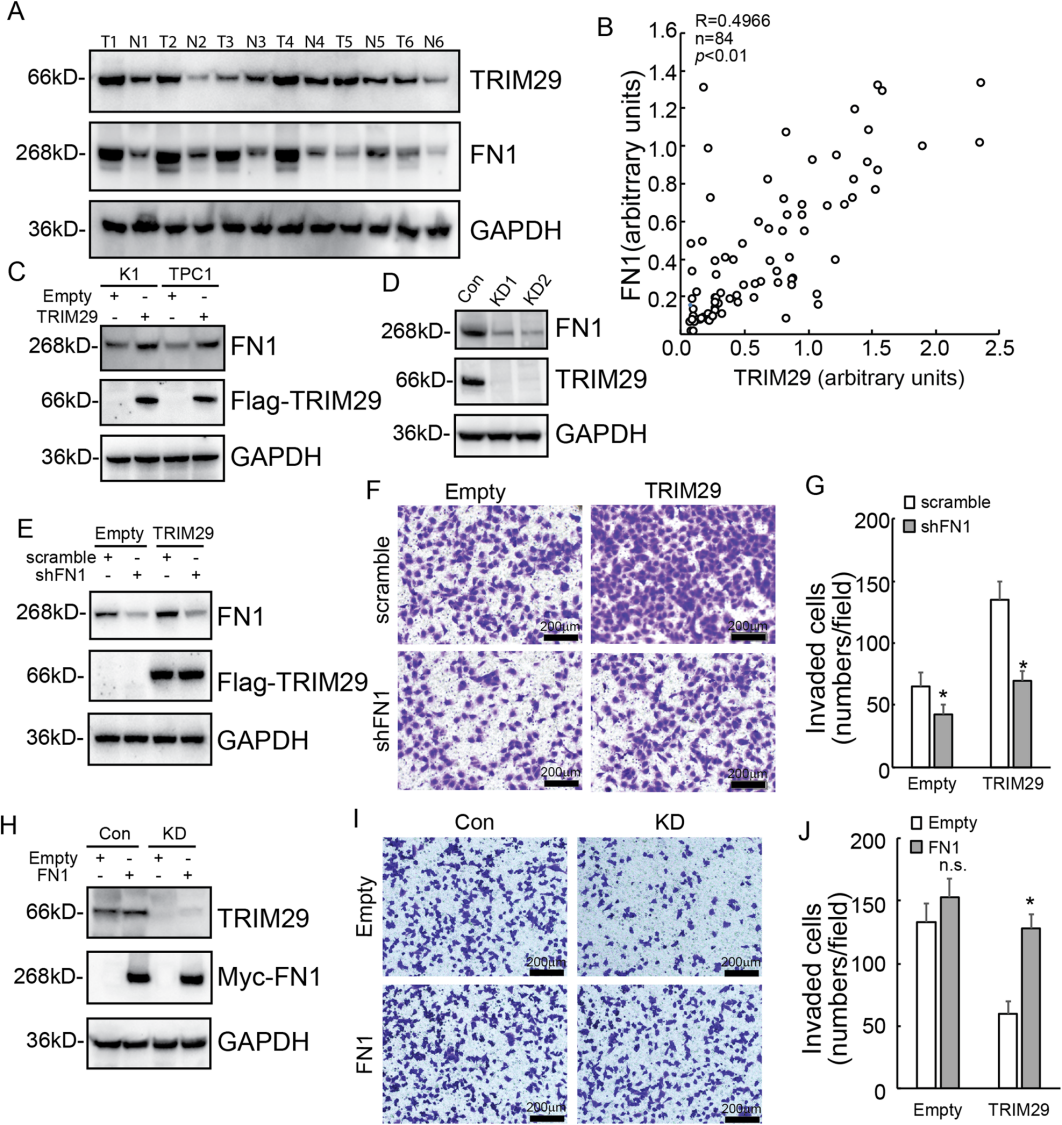
TRIM29 has a positive correlation with FN1, and affects invasive capacities of PTC cells by regulating FN1. **a** TRIM29 and FN1 protein levels were investigated using western blot in paired fresh papillary cancer (T) and adjacent noncancerous (N) tissues, and representative images were provided. **b** Scatter plot showing the correlation between TRIM29 and FN1 in papillary cancer tissues. Pearson’s coefficient tests were performed to assess statistical significance. **c** FN1 expression was investigated in K1 and TPC1 cells with ectopic TRIM29 overexpression. **d** FN1 expression was investigated in IHH4 cells with TRIM29 knockdown. **e**–**g** FN1 expression of control and TRIM29 overexpression TPC1 cells was knocked down using lentivirus containing shRNAs specific against FN1 (shFN1). FN1 knockdown efficiency was confirmed using western blot (**e**), invasion of cells were analyzed using Matrigel-coated Transwell, representative photographs were presented (**f**) and their numbers were counted (**g**). **h**–**j** Control or TRIM29 knockdown IHH4 cells were transfected with empty or FN1 containing eukaryotic expression vectors. FN1 expression was confirmed using western blot analysis (**h**), invasion of cells were analyzed using Matrigel-coated Transwell, representative photographs were presented (**i**) and their numbers were counted (**j**). The data are presented as the mean (SD) of three repeated experiments. *P < 0.05, n.s. not significant.

Using SILAC followed by spectrometry, we selected the differential protein expression and fibronectin 1 (FN1) was screened as a significantly downregulated molecule by TRIM29 knockdown in IHH4 cells. Western blot was performed in 84 paired thyroid tissues, both TRIM29 and FN1 protein expression was significantly increased in papillary cancer (T) tissues, when compared with those in adjacent noncancerous (N) tissues (Fig. 2a). In addition, a positive correlation of TRIM29 and FN1 protein expression was observed in PTC tissues (Fig. 2b). Western blot confirmed that TRIM29 overexpression significantly increased FN1 expression in both K1 and TPC1 cells (Fig. 2c), while TRIM29 knockdown significantly decreased FN1 expression in IHH4 cells (Fig. 2d). FN1 expression was then knocked down using shRNAs specific against FN1 (shFN1) in TPC1 cells (Fig. 2e). Transwell assays exhibited that knockdown of FN1 significantly weakened the promotive effects on invasion of both control and TRIM29 overexpression TPC1 cells, especially significant in TRIM29 overexpression cells (Fig. 2f, g). On the contrary, FN1 was overexpressed in IHH4 cells (Fig. 2h). Overexpression of FN1 increased invasion of IHH4 cells with TRIM29 knockdown, while had no obvious effect in control IHH4 cells (Fig. 2i, j). These results indicated that TRIM29 promoted invasion of PTC cells by regulating the expression of FN1.

### TRIM29 alters FN1 mRNA stability through Ago2-dependent mechanism

**Fig. 3:**
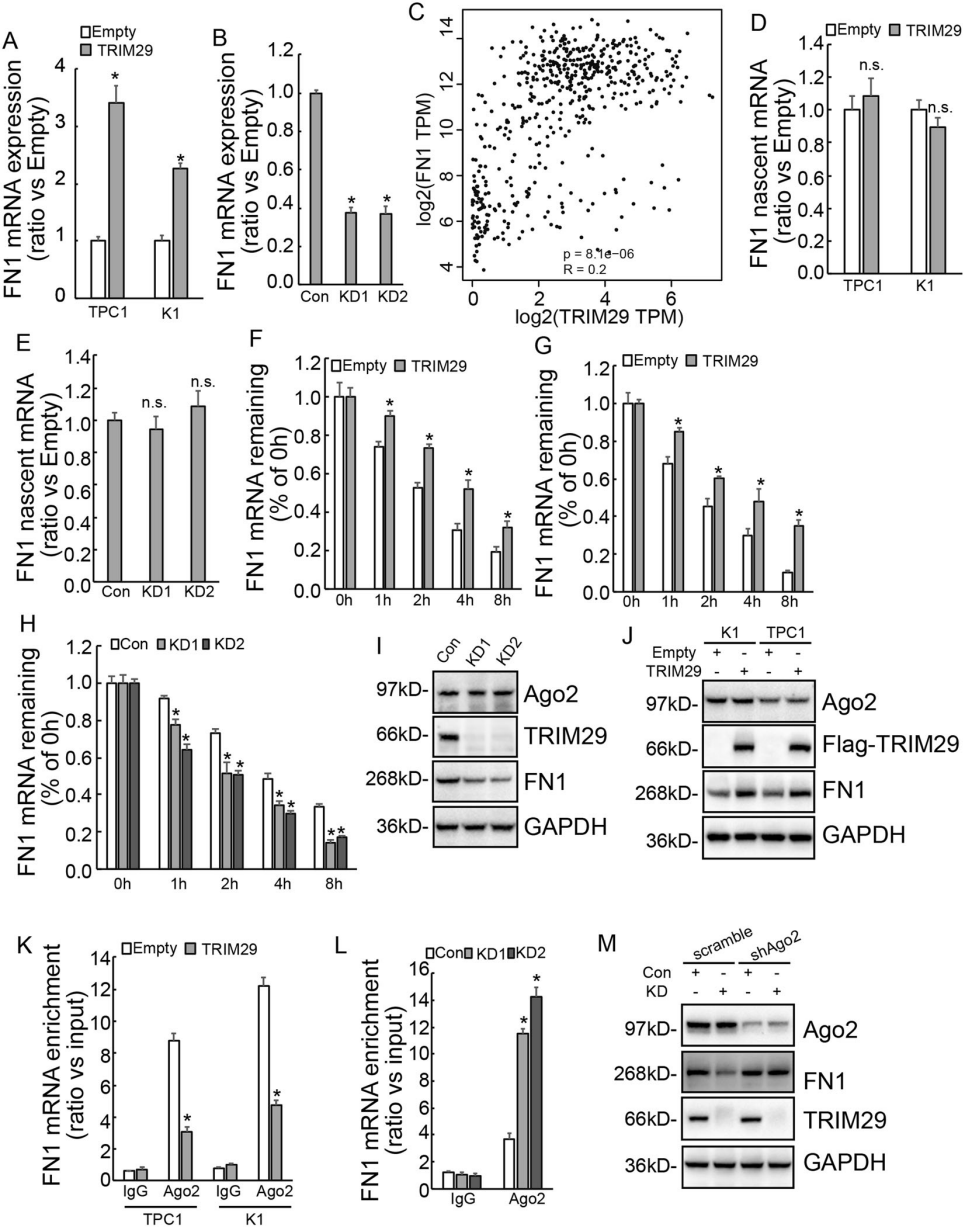
TRIM29 does not effect on de novo synthesis of FN1 mRNA, but alters FN1 mRNA stability through Ago2-dependent mechanism. **a** FN1 mRNA expression was analyzed using real-time RT-PCR in control or TRIM29 overexpression K1 and TPC1 cells. **b** FN1 mRNA expression was analyzed using real-time RT-PCR in control or TRIM29 knockdown IHH4 cells. **c** Scatter plot showing a positive co-expression of TRIM29 and FN1 in papillary cancer tissues according to database GEPIA. **d**, **e** Nascent RNA was labeled and isolated, and newly synthesized FN1 mRNA was analyzed using RT-PCR in control or TRIM29 overexpression K1 and TPC1 cells (**d**), also in control or TRIM29 knockdown IHH4 cells (**e**). **f**–**h** Actinomycin D was added for the indicated period to block RNA synthesis, and FN1 mRNA was analyzed using RT-PCR in control or TRIM29 overexpression K1 (**f**) and TPC1 (**g**) cells, also in control or TRIM29 knockdown IHH4 cells (**h**). **i**, **j** Ago2 expression was analyzed using western blot in control or TRIM29 knockdown IHH4 cells (**i**), also in control or TRIM29 overexpression K1 and TPC1 cells (**j**). **k**, **l** RIP was performed using the antibody against Ago2, enrichment of FN1 mRNA was analyzed using qRT-PCR in K1 and TPC1 cells (**k**), also in IHH4 cells (**l**). **m** Ago2 expression was knocked down in control or TRIM29 knockdown IHH4 cells, and FN1 expression expression was analyzed using western blot. The data are presented as the mean (SD) of three repeated experiments. *P < 0.05, n.s. not significant.

Real-time RT-PCR confirmed that overexpression of TRIM29 increased FN1 mRNA levels in both K1 and TPC1 cells (Fig. 3a), while knockdown of TRIM29 decreased FN1 mRNA levels in IHH4 cells (Fig. 3b). The database GEPIA also revealed a positive co-expression of TRIM29 and FN1 in thyroid cancer (Fig. 3c). Investigation of nascent mRNA demonstrated that neither TRIM29 overexpression in K1 and TPC1 cells (Fig. 3d) nor TRIM29 knockdown in IHH4 cells (Fig. 3e) altered de novo synthesis of FN1 mRNA. Furthermore, RNA synthesis inhibitor Actinomycin D was used to measure the half-life of FN1 mRNA, we found that remained FN1 mRNA was markedly increased in K1 and TPC1 cells with TRIM29 overexpression (Fig. 3f, g), while significantly decreased in IHH4 cells with TRIM29 knockdown (Fig. 3h). These data indicated that TRIM29 altered the stability of FN1 mRNA and regulated FN1 expression at the post-transcriptional level in PTC cells. As RNA-induced silencing complex (RISC) can bind to the 3′-untranslated region (UTR) of the target mRNA based on a partial miRNA–mRNA complementarity and regulate stability of target RNA, RIP was then performed using antibody against Ago2. Although neither knockdown (Fig. 3i) nor overexpression (Fig. 3j) of TRIM29 altered Ago2 expression obviously, TRIM29 overexpression significantly inhibited recruitment of Ago2 to FN1 mRNA in both K1 and TPC1 cells (Fig. 3k), while enrichment of FN1 mRNA by Ago2 was significantly augmented in IHH4 cells with TRIM29 knockdown (Fig. 3l). Ago2 expression was then knocked down in control or TRIM29 knockdown IHH4 cells, and it was showed that knockdown of Ago2 significantly rescued the FN1 expression suppressed by TRIM29 knockdown (Fig. 3m). Therefore, TRIM29 might regulate stability of FN1 mRNA via miRNA-mediated gene silencing (miRISC) in PTCs.

### TRIM29 regulates FN1 via miR-873-5p

**Fig. 4:**
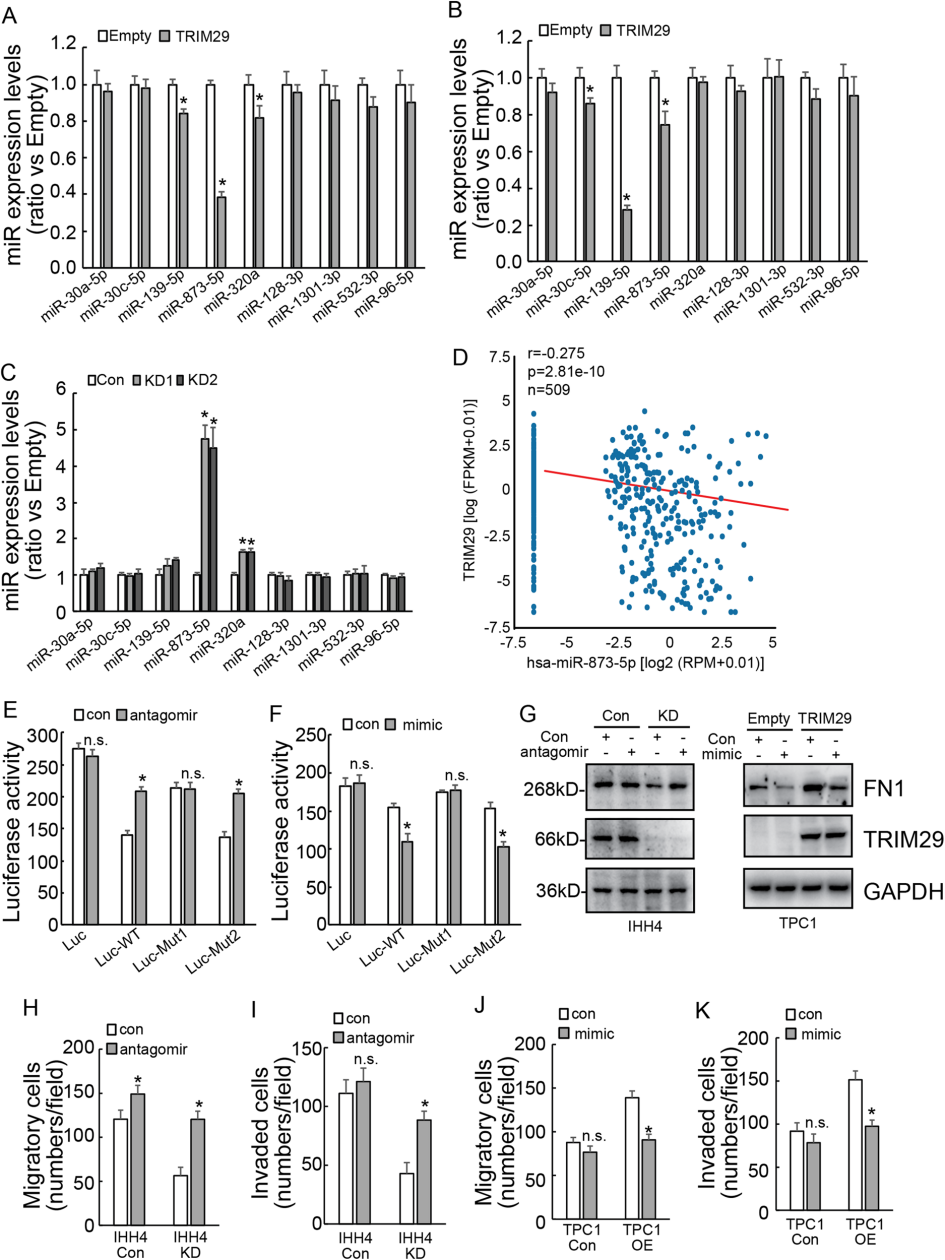
TRIM29 regulates FN1 via miR-873-5p in PTC cells. **a**–**c** Relative expression levels of miRNAs were detected by qRT-PCR in K1 cells (**a**), TPC1 cells (**b**) by TRIM29 overexpression and in IHH4 cells (**c**) by TRIM29 knockdown. **d** Scatter plot showing the positive co-expression between TRIM29 and has-miR-873-5p in papillary cancer tissues according to online database (starbase.sysu.edu.cn). **e**, **f** TPC1 cells were co-transfected with miR-873-5p antagomir (**e**) or mimic (**f**) and luciferase constructs containing 3′UTR of FN1 with WT and two miR-873-5p potential binding site mutation, luciferase activity was measured after 48 h of transfection. **g** control and TRIM29 overexpression TPC1 cells were transfected with control or miR-873-5p mimic, while control and TRIM29 knockdown IHH4 cells were transfected with control or miR-873-5p antagomir, FN1 expression was analyzed using western blot analysis. **h**, **i** control and TRIM29 knockdown IHH4 cells were transfected with control or miR-873-5p antagomir, migration (**h**) and invasion (**i**) of cells were analyzed using Matrigel-uncoated or Matrigel-coated Transwell, their numbers were counted. **j**, **k** control and TRIM29 overexpression TPC1 cells were transfected with control or miR-873-5p mimic, migration (**j**) and invasion (**k**) of cells were analyzed using Matrigel-uncoated or Matrigel-coated Transwell, their numbers were counted. The data are presented as the mean (SD) of three repeated experiments. *P < 0.05, n.s. not significant.

The bioinformatics analyses by combination of miRNA prediction databases and negative co-expression in thyroid cancer tissues were applied to explore the potential FN1 mRNA targeting miRNAs. A panel of miRNAs including miR-30a-5p, miR-30c-5p, miR-139-5p, miR-873-5p, miR-320a, miR-128-3p, miR-1301-3p, miR-532-3p, and miR-96-5p were screened out. qRT-PCR demonstrated that TRIM29 overexpression decreased miR-139-5p, miR-873-5p, and miR-320a expression in K1 cells (Fig. 4a), and decreased miR-30c-5p, miR-139-5p, and miR-873-5p in TPC1 cells (Fig. 4b). On the other hand, TRIM29 knockdown increased miR-873-5p and miR-320a in IHH4 cells (Fig. 4c). miR-873-5p was constantly regulated by TRIM29 in studied cells, indicating that TRIM29 might regulate stability of FN1 mRNA via regulation of miR-873-5p expression. Online database (starbase.sysu.edu.cn) also demonstrated a negative co-expression between TRIM29 and miR-873-5p in thyroid cancer tissues (Fig. 4d). Two potential binding sites for FN1 was predicted on the 3′UTR of FN1 mRNA (176–181 and 739–744). Luciferase constructs containing 3′UTR of FN1 with WT and miR-873-5p potential binding site mutation (Mut1 and Mut2, respectively) were then generated. Luciferase activity assays exhibited that miR-873-5p antagomir significantly increased (Fig. 4e), while miR-873-5p mimic decreased (Fig. 4f) the luciferase activity of reporter containing WT and the Mut2 FN1 3′UTR in TPC1 cells. However, neither miR-873-5p antagomir nor miR-873-5p mimic altered the luciferase activity of reporter construct containing Mut1 FN1 3′UTR (Fig. 4e, f). Western blot confirmed that miR-873-5p mimic weaken the promotion of FN1 expression by TRIM29 overexpression in TPC1 cells, while miR-873-5p antagomir partially restored the inhibition of FN1 expression by TRIM29 knockdown in IHH4 cells (Fig. 4g). In addition, miR-873-5p antagomir significantly increased migration (Fig. 4h) and invasion (Fig. 4i) of IHH4 cells with TRIM29 knockdown. miR-873-5p antagomir also increased migration (Fig. 4h), while unaltered invasion (Fig. 4i) of control IHH4 cells. On the contrary, miR-873-5p mimic significantly decreased migration (Fig. 4j) and invasion (Fig. 4k) of TPC1 cells with ectopic TRIM29 expression. miR-873-5p mimic demonstrated no obvious effects on migration (Fig. 4j) and invasion (Fig. 4k) of control TPC1 cells. Collectively, these data indicated that TRIM29 regulated FN1 expression, as well as migration and invasion of PTC cells via regulation of miR-873-5p expression.

### TRIM29 inhibits premiR-873 cleavage by suppressing recruitment of Dicer and TRBP

**Fig. 5:**
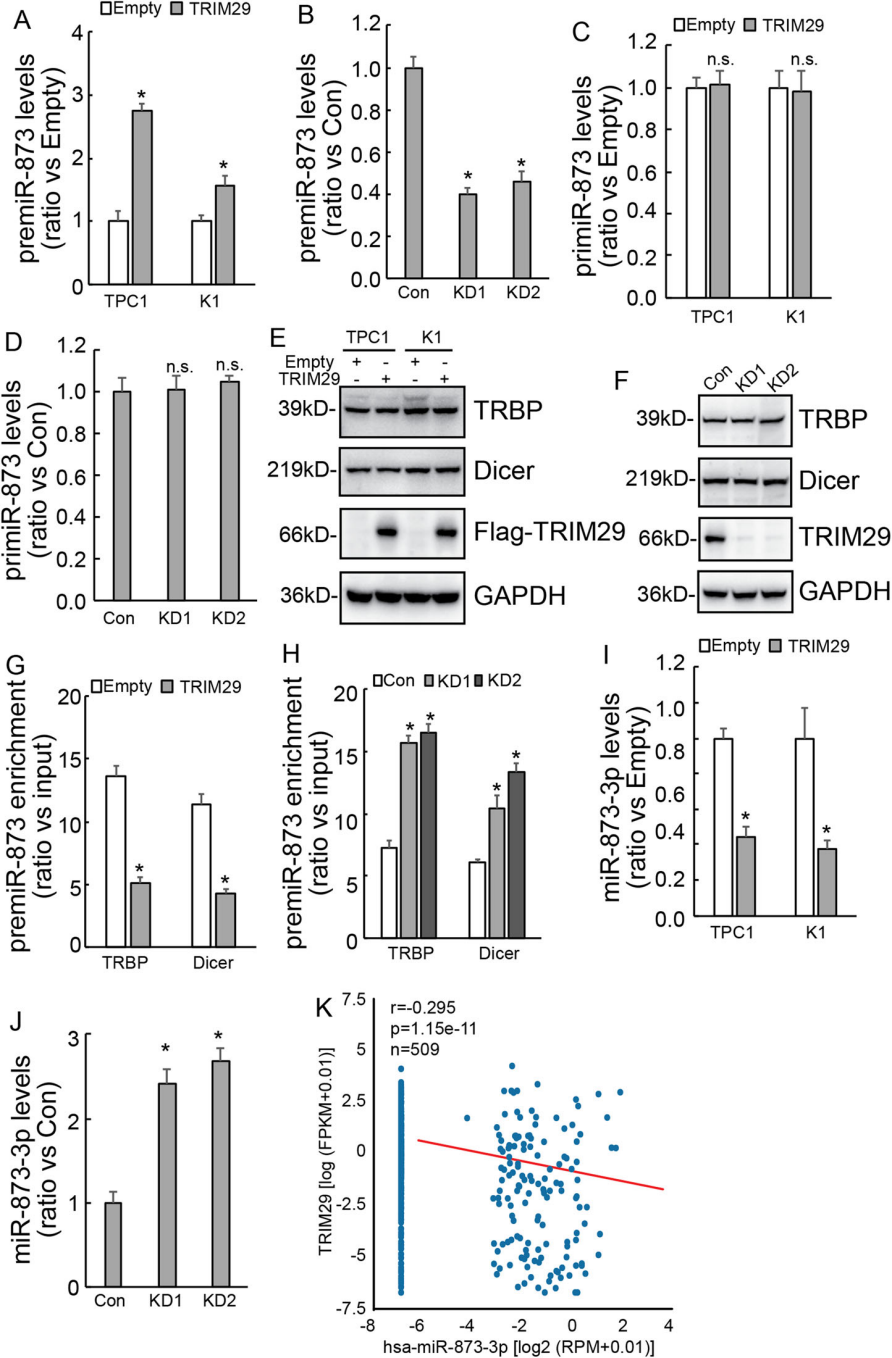
TRIM29 inhibits premiR-873 cleavage by suppressing it recruiting Dicer and TRBP. **a**, **b** premiRNA-873 expression levels were analyzed using real-time RT-PCR in control or TRIM29 overexpression K1 and TPC1 cells (**a**), also in control or TRIM29 knockdown IHH4 cells (**b**). **c**, **d** primiRNA-873 expression levels were analyzed using real-time RT-PCR in control or TRIM29 overexpression K1 and TPC1 cells (**c**), also in control or TRIM29 knockdown IHH4 cells (**d**). **e**, **f** Dicer and TRBP expression were investigated in K1 and TPC1 cells with ectopic TRIM29 overexpression (**e**), also in IHH4 cells with TRIM29 knockdown (**f**). **g**, **h** RIP was performed using the antibody against TRBP and Dicer, enrichment of premiR-873 was analyzed using qRT-PCR in TPC1 cells (**g**), and IHH4 cells (**h**). **i**, **j** Relative expression levels of miR-873-3p was also etected by qRT-PCR in K1 and TPC1 cells (**i**) by TRIM29 overexpression and in IHH4 cells (**j**) by TRIM29 knockdown. **k** Scatter plot also showing the negative co-expression between TRIM29 and has-miR-873-3p in papillary cancer tissues according to online database (starbase.sysu.edu.cn). The data are presented as the mean (SD) of three repeated experiments. *P < 0.05, n.s. not significant.

Biogenesis of mature miRNAs was known as a sequential and complex process, including cleavage of long primiRNAs by nuclear RNase III Drosha to release 60- to 70-nt hairpin premiRNAs, and subsequent cleavage of premiRNAs by cytoplasmic RNase III Dicer to generate their mature length. To explore the potential mechanisms underlying regulation of miR-873-5p by TRIM29, we analyzed the levels of premiR-873 and primiR-873 using qRT-PCR in PTC cells. Contrary to downregulation of miR-873-5p by TRIM29 overexpression (Fig. 4a, b) and upregulation of miR-873-5p by TRIM29 knockdown (Fig. 4c), TRIM29 overexpression increased premiR-873 in TPC1 and K1 cells (Fig. 5a) and TRIM29 knockdown decreased premiR-873 expression in IHH4 cells (Fig. 5b). Neither TRIM29 overexpression in TPC1 and K1 cells (Fig. 5c) nor knockdown in IHH4 cells (Fig. 5d) altered the expression of primiR-873. These data indicated that TRIM29 might inhibit cleavage of premiR-873 to generate mature miRNAs. Given the complex of the RNase Dicer and Tar RNA-binding protein (TRBP) play an important role in premiRNA cleavage, western blot was performed to investigate their expression. Neither TRBP nor Dicer expression was altered by TRIM29 overexpression in K1 and TPC1 cells (Fig. 5e) or knockdown in IHH4 cells (Fig. 5f). However, RIP showed that recruitment of Dicer and TRBP to premiR-873 was obviously suppressed by TRIM29 overexpression in TPC1 cells (Fig. 5g), while enhanced by TRIM29 knockdown in IHH4 cells (Fig. 5h). These data indicated that TRIM29 might suppress cleavage of premiR-873. Since cleavage of premiR-873 also generates miR-873-3p except for miR-873-5p, to clarify regulation of premiR-873 cleavage by TRIM29, miR-873-3p expression was then analyzed. Similar like miR-873-5p (Fig. 4a–c), miR-873-3p was decreased by TRIM29 overexpression in TPC1 and K1 cells (Fig. 5i), while increased by TRIM29 knockdown in IHH4 cells (Fig. 5j). Online database (starbase.sysu.edu.cn) also showed a negative co-expression between TRIM29 and miR-873-3p in thyroid cancer tissues (Fig. 5k). These results therefore indicated that TRIM29 regulates cleavage of premiR-873 to generate mature miR-873-5p and miR-873-3p.

### LncRNA CYTOR is implicated in regulation of FN1 by TRIM29

**Fig. 6:**
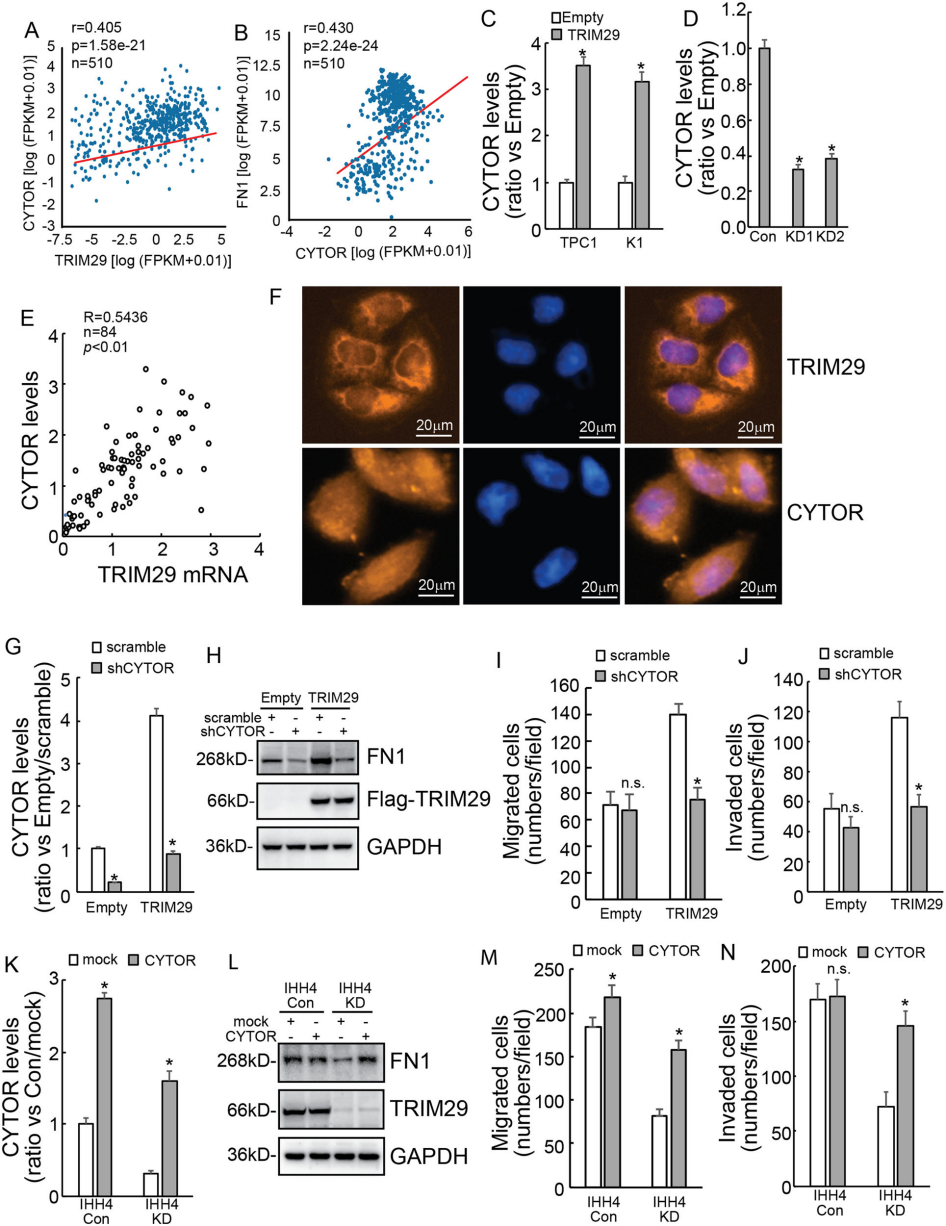
LncRNA CYTOR is involved in regulation of FN1 by TRIM29. **a**, **b** Scatter plot showing the positive co-expression between CYTOR and TRIM29 (**a**), FN1 (**b**) in papillary cancer tissues according to online database (starbase.sysu.edu.cn). **c**, **d** lncRNA CYTOR levels were analyzed using qRT-PCR in control or TRIM29 overexpression K1 and TPC1 cells (**c**), also in control or TRIM29 knockdown IHH4 cells (**d**). **e** Scatter plot showing the correlation between TRIM29 mRNA and lncRNA CYTOR in papillary cancer tissues. Pearson’s coefficient tests were performed to assess statistical significance. **f** The cellular distribution of TRIM29 and lncRNA CYTOR in IHH4 cells was investigated using immunofluorescence and FISH staining, respectively. **g** lncRNA CYTOR expression of TPC1 cells was knocked down using lentivirus containing shRNAs specific against lncRNA CYTOR (shCYTOR) with or without TRIM29 overexpression. **h**–**j** FN1 expression was analyzed using western blot in both control and ectopic TRIM29 expression TPC1 cells by CYTOR knockdown (**h**), metastatic, and invasive capacity were analyzed using Matrigel-uncoated or Matrigel-coated Transwell, the cell numbers were counted (**i**–**j**). **k** lncRNA CYTOR expression of IHH4 cells was overexpressed using vectors pcDNA3.1 with or without TRIM29 knockdown. **l**–**n** FN1 expression was analyzed using western blot in both control and TRIM29 knockdown IHH4 cells by CYTOR overexpression (**l**), metastatic and invasive capacity were analyzed using Matrigel-uncoated or Matrigel-coated Transwell, the cell numbers were counted (**m**, **n**).The data are presented as the mean (SD) of three repeated experiments. *P < 0.05, n.s. not significant.

RNA sequence demonstrated that several long non-coding RNAs (LncRNAs) were downregulated by TRIM29 knockdown, and LncRNA CYTOR was screened as online database exhibited that CYTOR was positively co-expressed with TRIM29 (Fig. 6a) and FN1 (Fig. 6b) in thyroid cancer tissues. We then verified that CYTOR expression was upregulated with TRIM29 overexpression in both K1 and TPC1 cells (Fig. 6c), while was downregulated with TRIM29 knockdown in IHH4 cells (Fig. 6d). We further confirmed the positive correlation of TRIM29 and CYTOR expression in 84 paired PTC tissues (Fig. 6e). The cellular distribution of TRIM29 and lncRNA CYTOR was also investigated using immunofluorescence and FISH staining, respectively. Immunofluorescence staining demonstrated that TRIM29 was mainly distributed in cytoplasm of IHH4 cells, FISH demonstrated that CYTOR was distributed in both nucleus and cytoplasm of IHH4 cells (Fig. 6f). Then, CYTOR expression was knocked down using shRNAs in TPC1 cells with or without TRIM29 overexpression (Fig. 6g). Western blot exhibited that CYTOR knockdown markedly decreased FN1 expression in both control and ectopic TRIM29 expression TPC1 cells (Fig. 6h). CYTOR knockdown significantly weakened the promotive effect of TRIM29 on migration (Fig. 6i) and invasion (Fig. 6j) of TPC1 cells with TRIM29 overexpression, while had no obvious effect in control TPC1 cells. To further clarify the regulatory role, CYTOR was also overexpressed in IHH4 cells with or without TRIM29 knockdown (Fig. 6k). Western blot demonstrated that CYTOR overexpression markedly increased FN1 expression, especially in TRIM29 knockdown IHH4 cells (Fig. 6l). CYTOR overexpression significantly promoted migration (Fig. 6m) and invasion (Fig. 6n) of IHH4 cells with TRIM29 knockdown, while demonstrated no obvious effect on invasion rather than migration in control IHH4 cells. Collectively, these results demonstrated that TRIM29 regulates FN1 expression, as wells as migration and invasion of PTC cells via CYTOR.

### TRIM29 suppresses pre-miR873 cleavage via upregulation of CYTOR

**Fig. 7:**
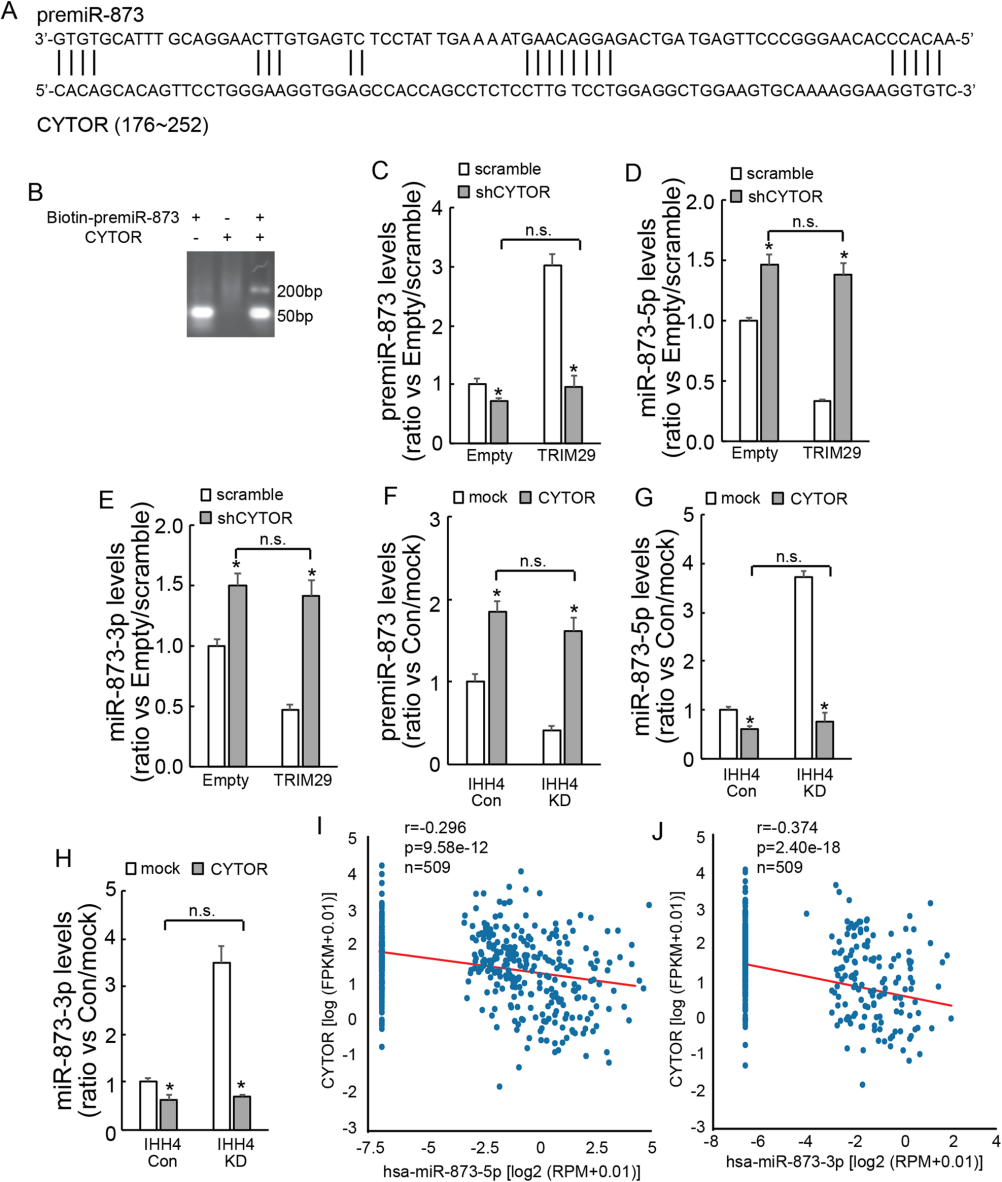
TRIM29 suppresses pre-miR873 cleaving to generate mature miRNAs via upregulation of CYTOR. **a** The abridged general view of potential binding sites between premiR-873 and CYTOR was showed according to online tool LncTar. **b** biotin-labeled premiR-873 was coincubated with CYTOR fragment to confirm their interaction by RNA EMSA. **c**–**e** The expression of premiR-873 (**c**), miR-873-5p (**d**), and miR-873-3p (**e**) were analyzed using qRT-PCR in TPC1 cells with or without TRIM29 overexpression by CYTOR knockdown. **f**–**h** The expression of premiR-873 (**f**), miR-873-5p (**g**), and miR-873-3p (**h**) were analyzed using qRT-PCR in IHH4 cells with or without TRIM29 knockdown by CYTOR overexpression. **i**, **j** Scatter plot showing the co-expression between CYTOR and hsa-miR-873-5P (**i**), hsa-miR-873-3P (**j**) in papillary cancer tissues according to online database (starbase.sysu.edu.cn). The data are presented as the mean (SD) of three repeated experiments. *P < 0.05, n.s. not significant.

An online tool LncTar predicted that CYTOR interacted with premiR-873 (Fig. 7a). RNA electrophoretic mobility shift assay (EMSA) using biotin-labeled premiR-873 demonstrated that shift migration of biotin-labeled premiR-873 was observed when coincubation with CYTOR fragment (Fig. 7b), confirming the interaction between premiR-873 and CYTOR. To analyze whether TRIM29 might regulate premiR-873 cleavage via upregulation of CYTOR, CYTOR was knocked down. Knockdown of CYTOR significantly reduced premiR-873 expression (Fig. 7c), while increased miR-873-5p (Fig. 7d) and miR-873-3p (Fig. 7e) expression in TPC1 cells. When CYTOR was knocked down, TRIM29 exhibited no obvious effects on either premiR-873 or mature miR-873-5p and miR-873-3p expression in TPC1 cells (Fig. 7c–e). In addition, CYTOR overexpression evidently increased expression of premiR-873 (Fig. 7f), but significantly decreased expression of mature miR-873-5p (Fig. 7g) and miR-873-3p (Fig. 7h). Online database (starbase.sysu.edu.cn) also exhibited that CYTOR was negativity co-expressed with miR-873-5p (Fig. 7i) and miR-873-3p (Fig. 7j) in thyroid cancer tissues. Thereby, these data indicated that TRIM29 regulates cleavage of premiR-873 via regulating CYTOR expression.

## Discussion

TRIM29 encodes for a member of the tripartite motif (TRIM) protein family, which generally function as E3 ubiquitin ligases as they contain a RING-finger domain^34^. Different from others, TRIM29 lacks the RING domain but has also been involved in broad range of cancers and cellular processes as well^35^. The abnormal expression of TRIM29 is controversial whether it promotes or suppresses cancer development. TRIM29 is often mentioned that it has been involved in the response to DNA damage through its regulation of p53 and potentially functioned as an oncogene that promotes tumor growth^35,36^. Several studies suggested that TRIM29 promoted progression of cervical cancer^18^, pancreatic cancer^19^, colorectal cancer^21^, gastric cancer^37^, and glioma^38^, while suppressed progression of hepatocellular carcinoma via Wnt/beta-catenin signaling pathway^22^. It also has been reported that TRIM29 promoted proliferation and survival of bladder cancer cells through NF-κB but inhibited TWIST1 and suppressed EMT in breast cancer^23^. In thyroid cancer, TRIM29 was observed as a oncogene to promote cell proliferation via PI3K/AKT signaling pathway and correlate with poor prognosis^24,25^. Partly consistent with the two reports, we also found TRIM29 expression is upregulated in papillary thyroid cancer tissues. However, the current study demonstrated that TRIM29 obviously promoted the migration/invasion ability but the effect on proliferation of thyroid cancer cells was limited.

Although PTC patients have an excellent prognosis, lymph node metastasis (LNM) is common, even approximately up to 30–80%^39^. Lateral lymph node metastasis (LLNM), local invasion, and distant metastasis could increase the risk of locoregional recurrence and decrease the survival^12,40^. A large part of thyroid cancers are <1 cm in size, which be defined as papillary thyroid microcarcinoma (PTMC)^3^. Therefore, we focused on the mechanisms underlying the invasiveness and metastasis regulated by TRIM29 in PTC. In this study, we found the positive correlation of TRIM29 and FN1 protein expression both in tissue and in cells, and what’s more, the inhibition of invasion with TRIM29 knockdown was rescued by ectopic FN1 expression in PTC cells. As a epithelial–mesenchymal transition (EMT) marker, FN1 has already been proven to promote PTC progression and cells migration and invasion in vitro^33^. These results indicated the mechanism by which TRIM29 regulates invasion and metastasis is accomplished through FN1.

The current study demonstrated that TRIM29 did not affect de novo FN1 mRNA synthesis but altered its stability in PTC cells. In addition, we found Ago2 was significantly recruited to FN1 mRNA in PTC cells. As Ago2 was a key component of RISC, which bind to miRNAs to guide post-transcriptional gene silencing either by destabilization of the mRNA or by translational repression^41,42^, we further identified that TRIM29 negatively regulated miR-873-5p expression, which was responsible for regulating the stability of FN1 transcript via targeting its 3′UTR region.

The miRISC is a multistep process beginning with transcription of primary miRNA (primiRNA), which is subsequently cleaved to generate premiRNA by the microprocessor complex containing DGCR8 and Drosha^43^. PremiRNAs are exported from the nucleus to the cytoplasm via Exportin-5 together with RanGTP^44^, and processed to generate a ~22-nt miRNA duplex by Dicer and TRBP^45^. Finally, One of the two strands is assembled into the RISC together with one of the Argonaute (Ago) proteins, and RISC can bind to 3′UTR region lead to translational inhibition and/or degradation^46^. In the case of premiR-873 can be cleaved to generate two mature miRNAs, miR-873-5p and miR-873-3p. Our findings demonstrated that primiR-873 levels were unaltered by TRIM29, while TRIM29 played opposite role between premiR-873 and mature miR-873s, indicating that TRIM29 might suppress processing of premiR-873 to generate miR-873-5p and miR-873-3p. The current study demonstrated that TRIM29 did not alter the expression of premiRs’ processing apparatus Dicer and TRBP, but did affect recruitment of Dicer and TRBP to premiR-873.

The current study demonstrated that TRIM29 suppressed cleavage of premiR-873 via upregulation of LncRNA CYTOR. LncRNA CYTOR usually plays promotive roles in many cancers. Consistent with the reports that LncRNA CYTOR was upregulated in colon cancer^30^ and gastric cancer^32^, our findings revealed CYTOR was not only upregulated by TRIM29 but also had a positive correlation with TRIM29 both in PTC tissues and cells for the first time.

In conclusion, our study reported that TRIM29 inhibited miR-873-5P biogenesis via lncRNA CYTOR sponging premiR-873 to upregulate FN1 and promoted invasion of papillary thyroid cancer cells. This novel mechanism provides a new insight on understanding the invasion and potential therapeutic strategy of PTC.
